# Targeting the Cbl-b-Notch1 axis as a novel immunotherapeutic strategy to boost CD8+ T-cell responses

**DOI:** 10.3389/fimmu.2022.987298

**Published:** 2022-08-26

**Authors:** Giulia Monticone, Zhi Huang, Fred Csibi, Silvana Leit, David Ciccone, Ameya S. Champhekar, Jermaine E. Austin, Deniz A. Ucar, Fokhrul Hossain, Salome V. Ibba, A. Hamid Boulares, Nicholas Carpino, Keli Xu, Samarpan Majumder, Barbara A. Osborne, Christine Loh, Lucio Miele

**Affiliations:** ^1^ Department of Genetics, Louisiana State University Health Sciences Center, New Orleans, LA, United States; ^2^ Nimbus Therapeutics, Cambridge, MA, United States; ^3^ Department of Microbiology, Immunology and Molecular Genetics, University of California, Los Angeles, CA, United States; ^4^ Department of Biology, University of Virginia, Charlottesville, VA, United States; ^5^ Department of Interdisciplinary Oncology, Louisiana State University Health Sciences Center, New Orleans, LA, United States; ^6^ Department of Microbiology and Immunology, Stony Brook University, Stony Brook, NY, United States; ^7^ Department of Neurobiology and Anatomical Sciences, University of Mississippi Medical Center, Jackson, MS, United States; ^8^ Department of Veterinary and Animal Sciences, University of Massachusetts, Amherst, MA, United States

**Keywords:** adenosine, Cbl-b, immunotherapy, immunosuppression, Notch1

## Abstract

A critical feature of cancer is the ability to induce immunosuppression and evade immune responses. Tumor-induced immunosuppression diminishes the effectiveness of endogenous immune responses and decreases the efficacy of cancer immunotherapy. In this study, we describe a new immunosuppressive pathway in which adenosine promotes Casitas B-lineage lymphoma b (Cbl-b)-mediated Notch1 degradation, causing suppression of CD8+ T-cells effector functions. Genetic knockout and pharmacological inhibition of Cbl-b prevents Notch1 degradation in response to adenosine and reactivates its signaling. Reactivation of Notch1 results in enhanced CD8+ T-cell effector functions, anti-cancer response and resistance to immunosuppression. Our work provides evidence that targeting the Cbl-b-Notch1 axis is a novel promising strategy for cancer immunotherapy.

## Introduction

Tumor-induced immunosuppression is a hallmark feature of cancer which allows tumors to evade immune surveillance and progress (1). This is a major challenge for endogenous anti-cancer immune responses as well as for the successful application of cancer immunotherapies (2). Therefore, there is a critical need for novel cancer immunotherapies that can not only boost the immune response, but also overcome tumor-induced immunosuppression.

Tumors achieve immunosuppression in different ways, including production of immunosuppressive molecules, recruitment of suppressive immune cells and formation of physical barriers to immune infiltration (3). Among these strategies, overproduction of adenosine, an ATP metabolite, plays a major part in suppressing immune responses in the tumor microenvironment (4). Adenosine modulates the immune response by activating transmembrane G-protein coupled receptors (GPCRs) expressed on the membrane of immune cells (5, 6). Several studies have shown that adenosine suppresses CD8+ T-cells by activating the adenosine A2A receptor (A2AR) and blocking A2AR with selective antagonists results in enhanced anti-cancer immune responses (4, 7–9). Our group has shown that A2AR activation leads to downregulation of Notch1, a key regulator of T-cell effector functions (7). Notch1 signaling is triggered by T-cell receptor (TCR) activation and modulates T-cell effector functions by regulating proliferation and production of cytokines, including γ-interferon (IFN-γ) and Granzyme B (GNZB) (10–14). In line with the role of Notch1 in stimulating effector T-cell functions, exogenous expression of Notch1 in CD8+ T-cells enhances anti-cancer T-cells responses and render T-cells resistant to immunosuppression in the tumor microenvironment of lung carcinoma and thymoma (15). On the contrary, inhibition of Notch1 activation with gamma-secretase inhibitors (GSI) has been shown to suppress T-cell activation, reduce proliferation and cytokine production (16–18). These findings suggest that Notch1 is a potential target to control tumor-induced immunosuppression.

Notch receptors are heterodimeric transmembrane proteins consisting of a large extracellular domain for ligand binding and a transmembrane domain. Upon binding to ligands presented on adjacent cells, the extracellular subunit dissociates from the transmembrane subunit, which the undergoes proteolytic cleavages mediated by ADAM10 and gamma-secretase, respectively. This releases the Notch intracellular domain (NICD) which will translocate into the cell nucleus, form a complex with co-activators and activate transcription of target genes (19, 20). Notch signaling can also be regulated in a ligand-independent manner (21–23). Ligand-independent endocytic regulation of Notch is of particular importance in *Drosophila* (24–26), as well as, in mammalian T-cells, where TCR activation triggers ligand-independent activation of Notch1 (10, 12, 13). Several studies have reported that different ubiquitin ligases are involved in the ligand-independent regulation of Notch in *Drosophila* (24, 26, 27). However, it is yet unknown which and how ubiquitin ligases are involved in regulating Notch1 in T-cells.

Casitas B-lineage lymphoma b (Cbl-b) is an E3 ubiquitin ligase that has been identified as an important negative regulator of TCR signaling cascade. Cbl-b controls the threshold of T-cell activation and T-cell anergy (28, 29). Cbl-b has been implicated in the degradation of surface receptors in coordination with the Tyr-phosphatase Suppressor of T-cell receptor signaling 1 (STS-1 or UBASH3B), another negative regulator of TCR signaling (30–32). Cbl-b deficiency in T-cells has been associated with enhanced T-cell function and anti-tumor potential. Indeed, Cbl-b deficient T-cells have a lower activation threshold and can be stimulated in the absence of CD28 co-stimulation (29). Mice lacking Cbl-b reject tumors because of increased T-cell and NK cell immune responses (33–35) and depletion of Cbl-b inhibits exhaustion in CD8+ T-cells and CAR-T cells (36).

Tumor-induced immunosuppression, including adenosine-mediated suppression of T-cells, poses a major limitation to the efficacy of cancer immunotherapy. To identify new effective immunotherapeutic strategies to overcome tumor-induced immunosuppression, here we investigated the immunosuppressive pathway regulating Notch1 downstream of A2AR and strategies to target this pathway to enhance T-cell anti-tumor responses. Our results identified a new regulatory axis in which adenosine, *via* A2AR, promotes Cbl-b-mediated Notch1 degradation, causing immunosuppression in CD8+ T-cells. We showed that genetic KO and pharmacological inhibition of Cbl-b prevents Notch1 degradation in response to adenosine and reactivates its signaling, thus resulting in enhanced CD8+ T-cell effector functions, anti-cancer response and resistance to immunosuppression. Our findings indicate that the Cbl-b-Notch1 axis is a novel promising target for cancer immunotherapy.

## Materials and methods

### Mice

C57BL/6 and FVB mice (6-8 week-old) were purchased from The Jackson Laboratory (Bar Harbor, ME) and housed in specific pathogen-free conditions in a 12h light/12h dark cycle with food and water available ad libitum. For isolation of CD8+ T-cells, spleens and lymph nodes were aseptically harvested post-euthanasia. For the orthotopic TNBC models 2x10^6 million C0321 or M-WNT cells were injected into the 4th mammary fat pad of C57BL/6 or FVB mice, respectively. For the syngeneic colon adenocarcinoma model, 2.5x10^5 MC-38 were injected subcutaneously in C57BL/6 mice. All experiments involving mice were approved by the Institutional Animal Care and Use Committee (IACUC) at LSUHSC (New Orleans, LA).

### Cell lines

C0321 and M-WNT TNBC cell lines were developed as described in Zhang, (37), Zhang, (38) and Dunlab, (39). MC-38 colon adenocarcinoma cell line and 293T cells were purchased from ATCC (Manassas, VA). C0321 and 293T cells were cultured in DMEM and M-WNT and MC-38 cells in RPMI. Both media were supplemented with 10% fetal bovine serum, 4 mM L-Glutamine, 50 U/ml penicillin and 50 µg streptomycin (Gibco).

### Primary CD8+ T-cells

CD8+ T-cells were aseptically isolated from the spleen and lymph nodes of C57BL/6 or FVB (6-8 weeks old) mice using the negative selection Easysep mouse CD8+ T-cell isolation kit (StemCell Technologies) according to the manufacturer’s instructions. Spleens and lymph nodes from Cbl-b KO (*Cbl-b ^-/-^*) were kindly provided by Dr. J. Chiang [NCI-NIH; (29)] and STS-1/STS-2 double KO (*STS-1 ^-/-/^STS-2 ^-/-^*) by Dr. N. Carpino [Stony Brook University; (31)]. CD8+ T-cells were cultured in RPMI supplemented with 10% fetal bovine serum, 4 mM L-Glutamine, 50 U/ml penicillin, 50 µg streptomycin and 50 µM 2-mercaptoethanol. The cells were activated in plates coated with anti-mouse CD3ϵ and anti-mouse CD28 antibodies (1 µg/ml, BD Biosciences) for up to 72h.

### CRISPR-Cas9 Notch1 KO CD8+ T-cells

Mouse pan T-cells were isolated from C57BL/6 mouse spleens and immediately subjected to electroporation. crRNAs targeting the gene of interest were mixed with tracrRNA in 1:1 ratio and annealed at 95°C for 5min. 300pmol of crRNA:tracrRNA (IDT) duplex were combined with 100pmol Cas9 (IDT) and incubated at room temperature for at least 15min to generate Cas9/gRNA RNP. 1-5 x 10^6 mouse T cells were collected by centrifuging at 300g for 5min and re-suspended in 100ul mouse T-cell Nucleofector^®^ Solution (Lonza). Cell suspensions were mixed with Cas9/gRNA RNP and transferred in cuvettes for electroporation with the Nucleofector 2b device (Lonza). Program X-001 was used for electroporation. 500ul Pre-warmed IL-2-containing (10ng/ml) complete T-cell medium (RPMI with 10% FBS, 2 mM GlutaMAX, 1 mM sodium pyruvate, 0.1 mM nonessential amino acids, 55 μM β-mercaptoethanol, 100 U/ml penicillin,100 μg/ml streptomycin, and 10 mM Hepes) was added immediately into the cuvette after electroporation and cells were gently transferred to a 12-well plate prefilled with 1.5ml pre-warmed complete T-cell medium containing IL-2 in each well and cultured in 37°C with 5% CO2. Mouse T-cells were then activated with anti-CD3/anti-CD28 for 5h after electroporation, in complete T-cell medium supplemented with IL-2. Transfection efficiency was evaluated 3 days post-electroporation and positively edited T-cells were sorted on the basis of surface Notch expression level. Cells were then cultured in complete T-cell medium containing IL-2 and IL-7 (10ng/ml) for a week. We used the following crRNA targeting mouse Notch1, 5’-TTGAGATGCTCCCAGCCAAG-3’, which was validated in Sawangarun et al. (40).

### Tumor-derived organoids and imaging

Organoids were derived from C0321, M-WNT or MC-38 tumors in FVB and C57BL/6 mice. To establish organoids cultures, tumors were minced and digested at 37°C in FBS-free DMEM/F12 Glutamax containing 1mg/ml type IV collagenase (Gibco). Digested tumors were passed through a 100 μm and a 70 μm strainer to isolate organoids of 70-100 μm in size. The organoids were resuspended in type I rat tail collagen gel (Gibco) and plated in 8-well chambered coverslips (µ-slide 8 well, Ibidi). The organoids cultures were hydrated with DMEM/F12 Glutamax supplemented with 5% FBS, 50 U/ml penicillin and 50 µg streptomycin. Treatments were added directly into the medium after plating the organoids-collagen cultures. Cells in organoids were stained using CellTracker™ Red CMTPX (cytosol, Invitrogen) and Hoechst 33342 (nucleus, BD Biosciences), and dead cells were labelled using a cell membrane impermeable nucleic acid dye, NucGreen™ Dead 488 ReadyProbes™ (Invitrogen). Infiltrating CD8+ T-cells were stained in organoids using rat anti-mouse CD8α PE-conjugated antibody (Clone 53-6.7, BD biosciences). For CD8-Notch1 co-localization experiments, organoids were fixed in 2% formalin, permeabilized in 0.2% Triton and block with 2.5% goat serum and Fc block (BD Biosciences). CD8+ T-cells were stained with rat anti-mouse CD8α PE-conjugated antibody (Clone 53-6.7, BD biosciences) and Notch1 with rabbit anti-Notch1 (D6F11, Cell Signaling Technology) primary antibody, and goat anti-rat Texas Red (Invitrogen), goat anti-rabbit Alexa Fluor 488 (Invitrogen) secondary antibodies, respectively. Cell nuclei were stained using DAPI.

Organoids were imaged at day 0-6 using a BZ-x800 microscope (Keyence) with 4x, 20x or 60x objectives. To measure the size of organoids over time, bright field images of a given organoid were taken at day 0, 4 and 6 of culture using the multi-point tool of BZ-x800, which allows to save a specific position in the culture plate. The area of the organoids was measured using ImageJ. To quantify cell death in organoids, the area positive for dead cell staining was measured and normalized by the total area of organoids using the BZ-x800 analyzer software; or the fluorescence intensity of the dead cell staining was measured and normalized by the area of the organoids using ImageJ. The same method was used to quantify the infiltration of CD8+ T-cells, by measuring the CD8+ area/fluorescence intensity and normalizing by the total organoid area. To quantify the co-localization between Notch1 and CD8, Notch1 fluorescence intensity was measured within the CD8+ areas of at least three different images per sample using ImageJ. Full co-localization fluorescence intensity was set to a value of 100 and the fluorescence intensity values obtained were expressed in percentages accordingly. Images were processed using the BZ-x800 analyzer software. For clarity, we reduced the threshold intensity of Notch1 staining in organoids to highlight Notch1 staining in CD8+ T-cells, which is more focused and intense compared to the staining in surrounding cancer and stroma cells. The threshold-adjusted images are presented in **Figure 6C**, whereas the original images are presented in **Supplementary Figure 7B**.

For flow cytometry analysis, organoids were dissociated into single cells at 37°C in FBS-free DMEM/F12 Glutamax containing 1mg/ml type IV collagenase (Gibco). Cells were stained for viability using the Fixable Viability Stain 780 (FVS780, BD biosciences) for 10min in PBS at RT, surface stained with anti-CD45 (FITC rat anti-mouse, clone 30-F11, BD biosciences), CD3 (BV421 hamster anti-mouse, clone 145-2C11 RUO, BD biosciences), CD8 (BV711 rat anti-mouse, clone 53-6.7 RUO, BD biosciences) in PBS at 4°C, fixed with 2% formaldehyde for 20min at 4°C, permeabilized using the Perm/Wash buffer of the Transcription Factor Buffer Set (BD biosciences) and intracellularly stained with anti-IFN-gamma (PE-CF594 rat anti-mouse, clone XMG1.2 RUO, BD biosciences). Flow cytometric analysis was performed using Gallios cytometer (Beckman Coulter). Results were analyzed using Kaluza software (Beckman Coulter) and CD8+ IFNg+ cells were identified by gating for live cells, CD45+ and CD3+.

### Compounds and potency studies

A2AR agonist, CGS-21680, and antagonist, ZM-241385, were purchased from Tocris. Cbl-b inhibitors, NTX-512, NTX-447 and NTX-307, were produced by Nimbus Therapeutics. TR-FRET was used to assess the potency of the compounds. Briefly, recombinant human Cbl-b (aa 36-427) was expressed in *E. coli*, purified and biotinylated *in vitro*. Recombinant human Src (aa 254-536)-Zap-70 (aa 281-297) fusion protein was expressed in *E. coli* and purified. Recombinant human UBE2D2(C85K) was expressed in *E. coli*, purified, ubiquitinated and BODIPY labelled *in vitro*. The compounds were dissolved in DMSO (typically at 10-20mM), and a ten-point half log dilution series was prepared using acoustic dispensing. The assay was performed by adding Cbl-b enzyme and Src-Zap/ATP (enzyme reaction) in the presence of UBE2D2(C85K)-Ub-FL-BODIPY, Streptavidin-Tb (binding reaction). The assay signal was measured at 520nm on an Envision plate reader, with reference signal at 620nm. Data was normalized using high and low assay controls: % Inhibition =100-(100*((high control) - unknown)/(high control - low control)). A 4-parameter dose-response equation was used to fit the normalized dose-response data and derive an IC50 for the compounds.

### Constructs and transfection

The constructs used were as follows: all HA-tagged Cbl-b constructs were a gift from Dr. Stanley Lipkowitz (41, 42) while the His-tagged ubiquitin construct was from Dr. Dirk Bohman (43). NTM and all Myc-tagged Notch1 deletion constructs have been previously described (44, 45). Lipofectamine 3000 (Invitrogen) was used to transfect 293T cells following the manufacturer’s instructions.

### Luciferase reporter assay

Luciferase assays were performed using the Dual Luciferase assay kit (Promega). 293T cells were transfected with Notch and Cbl-b constructs as indicated. All cells received a Hes-luciferase construct and a Renilla luciferase expression vector (pRL-CMV). Cells were cultured for 48 hours, harvested and lysates were made as per the manufacturer’s protocol. Reporter gene transcription was measured using a luminometer.

### Immunoprecipitation and western blot

Primary CD8+ T-cells were lysed in RIPA lysis buffer (Santa Cruz Biotechnology) for western blot or in Pierce IP lysis buffer (Thermo Fisher) for IP, supplemented with 1 mM Protease Inhibitor Cocktail (Thermo Fisher), 1 mM PMSF and 1 mM sodium orthovanadate. For IP, lysates were pre-cleared using Pierce Protein A/G Magnetic beads (Thermo Fisher) for 1 hour. Pre-cleared lysates were incubated over night at 4°C with end-over-end rotation with 2 µg of anti-Notch1 (D1E11, Cell Signaling) or anti-Cbl-b (G1, Santa Cruz Biotechnology) or rabbit IgG control antibody (Thermo Fisher). Pierce Protein A/G Magnetic beads (Thermo Fisher) were added and incubated for 1 hour at room temperature with end-over-end rotation. The beads were washed three times with lysis buffer and two times with deionized water at RT, resuspended in 2X Laemmli sample buffer (Biorad) and incubated at 95°C for 5 minutes to elute the immunoprecipitated proteins. For mass spectrometry, IP samples were prepared using Pierce MS-compatible Magnetic IP kit (Thermo Fisher) according to the manufacturer’s instructions.

293T cells were transfected with constructs as indicated, cultured for 48 hours and lysates were prepared using a lysis buffer containing 50 mM Hepes (pH 7.8), 1% NP-40, 250 mM NaCl, Pefabloc (0.125 mM, Fluka) and approximately 1 mg of protein was used per IP. For the ubiquitination assay, the proteosomal inhibitor lactacystin (20 μM, Kamiya Biomedical Corp.) was added to the culture media for 8 hours before preparing protein lysates with the above lysis buffer supplemented with N-Ethylmaleimide (25mM, Sigma). Lysates were pre-cleared using Protein G Sepharose beads (GE Healthcare) for 1 hour at 4°C and, depending upon the experiment, 4-6 µg of anti-Notch1 (C-20, Santa Cruz Biotechnology), anti-HA tag (Cell Signaling) or rabbit IgG control antibody (Santa Cruz Biotechnology) was added to pre-cleared lysates. After incubating on ice for 2 hours, 30 µl of Protein G Sepharose beads were added per tube and incubated for 1 hour at 4°C with end-over-end rotation to pull down antigen-antibody complexes. Beads were washed thoroughly with lysis buffer at RT, resuspended in 2X Laemmli sample buffer (Biorad) and incubated at 95°C for 5 minutes to elute the immunoprecipitated proteins.

For Western blotting, protein lysates were resolved on 4-15% or 7.5% SDS-PAGE gels (Biorad) and transferred on to PVDF membranes (Millipore). Blots were incubated overnight with primary antibody diluted in Intercept blocking buffer (Licor). The next day, blots were incubated with an appropriate HRP-conjugated secondary antibody for chemiluminescence detection, or with IRDye fluorescent secondary antibodies (Licor) for fluorescence detection, for 1 hour at RT. Proteins were visualized by developing the blots with ECL reagent (Biorad) or imaged on an Odyssey scanner (Licor). The following primary antibodies were used for Western blot in this study: anti-Notch1 (D1E11, Cell Signaling, or mN1A, Novus, or C-20, SCBT) for Notch1 full length and cleaved forms; anti-cleaved Notch1 Val1744 (D3B8, Cell Signaling or PA5-99448, Invitrogen-Thermofisher) for NICD; anti-Cbl-b (G1, Santa Cruz Biotechnology or 12781-1-AP, Proteintech); anti-STS-1 (19563-1-AP, Proteintech); anti-β-actin (AC-15, SCBT); anti-phospho-Tyr (PY99, STCB); anti-HA tag (Clone 6E2, Millipore).

### Mass spectrometry

Mass spectrometry analyses was performed by Dr. Samuel Mackintosh’s team as part of the IDeA National Resource for Quantitative Proteomics Voucher program. The samples were trypsin-digested and analyzed through data independent acquisition (DIA) quantitative proteomic platform in an Orbitrap Exploris 480 mass spectrometer.

### ELISA assay

Cytokine production was measured in supernatants of primary CD8+ T-cells activated and treated as explained above, using ELISA kits (Invitrogen) according to the manufacturer’s instructions.

### Proliferation assay

Proliferation was measured by labelling primary CD8+ T-cells with 1 μM carboxyfluorescein diacetate succinimidyl ester (CFSE, Thermo Fisher) for 10 min at 37°C before activation and treatments. CFSE fluorescence was measured by flow cytometric analysis on a Gallios cytometer (Beckman Coulter) and data analyzed using Kaluza software (Beckman Coulter).

### Statistics

Two-tailed unpaired Student’s *t*-test was used for pairwise comparisons between two groups and one-way ANOVA with Bonferroni correction for multiple comparisons was used for comparisons between multiple groups. P values ≤ 0.05 were considered significant.

## Results

### A2AR modulates Notch1 degradation and T-cell functions in CD8+ T-cells

**Figure 1:**
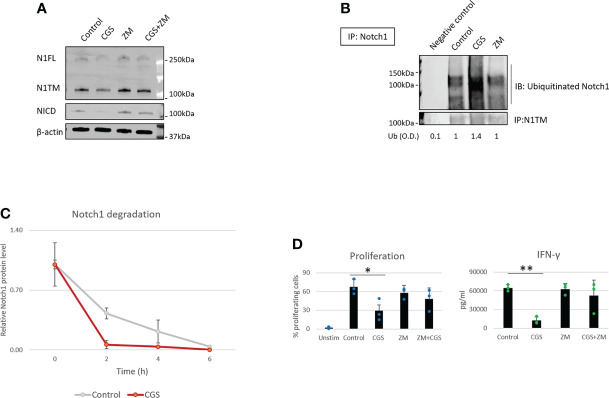
A2AR modulates Notch1 degradation and T-cell functions. Primary CD8+ T-cell isolated from the spleen and lymph nodes of C57B16 or FVB mice were stimulated with anti-CD3/CD28 antibodies and treated with vehicle (control, DMSO) or 1µM ZM-241385 (ZM) or 1µM CGS-21680 (CGS) or ZM+CGS, as indicated in the figure. **(A)** Protein levels of Notch1 full length (N1FL), Notch1 transmembrane (N1TM) and Notch1 transcriptionally active form (NICD) in primary activated CD8+ T-cells. **(B)** Ubiquitinated Notch1 protein levels from Notch1 immunoprecipitation in primary activated CD8+ T-cells. Densitometry (O.D.) results for ubiquitinated Notch1 normalized by IP:N1TM are shown below the panel. Negative control refers to samples immunoprecipitated using beads but not antibody. **(C)** Notch1 protein levels over time in primary activated CD8+ T-cells in the presence of the protein synthesis inhibitor Cycloheximide (CHX). The plot shows densitometry results of western blots. **(D)** Proliferation and production of IFN-γ in supernatants from samples of primary activated CD8+ T-cells. The graphs show averages ± standard deviation from three independent experiments. *p < 0.05, **p < 0.01, two tailed T-test with equal variance. Unstimulated cells (Unstim) and vehicle treated cells (control) were used as controls. β-actin was used to normalize densitometry values.

Our previous work showed that A2AR activation by adenosine downregulates Notch1 in CD8+ T-cells (7). However, how A2AR regulates Notch1 is still unknown. In our previous study, we did not observe effects of A2AR activation on Notch1 mRNA (7), suggesting that Notch1 downregulation may occur at the protein level. Therefore, we hypothesized that Notch1 downregulation by A2AR activation was the result of increased protein degradation. To test this idea, we isolated primary CD8+ T-cells from mouse spleens, activated with anti-CD3/CD28, and treated the cells with the selective A2AR agonist CGS-21680 (CGS), which mimics the effect of physiological adenosine and activates A2AR, and the selective A2AR antagonist, ZM-241395 (ZM), which blocks A2AR. We observed that Notch1 full length (N1FL), transmembrane Notch1 (N1TM) as well as the cleaved, transcriptionally active form of Notch1 (NICD) were downregulated in CD8+ T-cells treated with CGS, whereas the A2AR antagonist ZM rescued CGS-mediated downregulation (**Figure 1A**). To test whether Notch1 downregulation by CGS was the result of increased protein degradation, Notch1 was immunoprecipitated and ubiquitination was detected. We observed that CGS dramatically increased the ubiquitination of Notch1, compared to untreated and ZM-treated CD8+ T-cells (**Figure 1B**). To confirm that increased ubiquitination was related to more degradation, we treated CD8+ T-cells with the protein synthesis inhibitor Cycloheximide (CHX) and detected Notch1 protein levels at different time points. We found that Notch1 was decreased more rapidly in CGS treated CD8+ T-cells (**Figure 1C** and **Supplementary Figure 1**), further suggesting that A2AR activation promotes Notch1 degradation. Finally, we confirmed that A2AR activation results in immunosuppression. We observed that CGS decreased proliferation and IFN-gamma production, which is a transcriptional target of Notch1 (12, 13), in CD8+ T-cells (**Figure 1D**).

**Figure 2:**
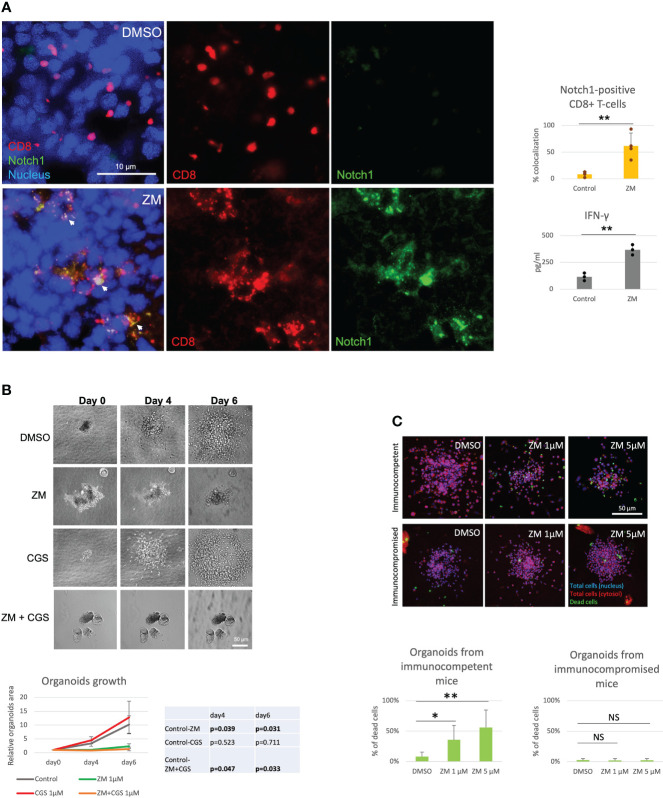
A2AR antagonist ZM enhances Notch1-positive T-cell anti-tumor activity. Organoids were derived from a syngeneic TNBC mouse model, C0321, in FVB mice or immunocompromised athymic Nu/Nu mice and treated with vehicle (control, DMSO) or 1µM ZM-241385 (ZM) or 1µM CGS-21680 (CGS) or ZM+CGS, as indicated in the figure. **(A)** Colocalization of Notch1 and CD8+ T-cells and production of IFN-gamma in organoids. **(B)** Growth of organoids measured over time. **(C)** Cancer cell death in organoids from immunocompetent *vs*. immunocompromised mice. Scale bars length is indicated above each bar (µm). The graphs show averages ± standard deviation from ≥ 10 organoids from at least three independent experiments. *p < 0.05, **p < 0.01, two tailed T-test with equal variance. NS, non-significant.

Several studies have shown that A2AR blockade with selective antagonists results in enhanced T-cell function and anti-tumor activity (4, 8, 9). Consistently, we observed that the A2AR antagonist ZM reverses Notch1 degradation and CGS-induced suppression of T-cell functions (**Figures 1A–D**), indicating that rescuing Notch1 from degradation promotes activation of T-cell function including proliferation and cytokine secretion. We also asked if restoring Notch1 in CD8+ T-cells, by blocking A2AR, promotes anti-tumor immunity. To test this idea, we generated tumor-derived organoids from a syngeneic Triple-Negative-Breast Cancer (TNBC) model, C0321 (37, 38), treated with ZM or CGS. Tumor-derived organoids are clusters of cells which contain all cell types present in the original tumor, including cancer cells, infiltrating immune cells and stroma cells and are a reliable *ex vivo* system that recapitulates the features of the original tumor and its microenvironment (46, 47). Remarkably, we found that more CD8+ T-cells were positive for Notch1 in ZM-treated organoids than in controls (**Figure 2A**) and ZM-treated organoids displayed increased IFN-gamma production, higher infiltration and clusters of CD8+ T-cells surrounding cancer cells, instead of the dispersed organization of T-cells observed in control organoids (**Figure 2A** and **Supplementary Figure 2A**). This pattern of Notch1 positive CD8+ T-cells associated with increased anti-tumor effect, as ZM treatment significantly suppressed tumor organoid growth and increased cancer cell death compared to control and CGS-treated organoids (**Figures 2B, C**). In addition, ZM was only effective in organoids derived from immunocompetent mice, but not from immunocompromised athymic Nu/Nu mice, consistent with the finding that the anti-tumor effect of ZM is immune-mediated (**Figure 2C** and **Supplementary Figure 2B**; 4, 8, 9). Taken together, our results indicate that A2AR activation suppresses CD8+ T-cells function, at least in part, through promoting Notch1 degradation, and A2AR blockade restores Notch1, T-cell function and anti-tumor potential.

### A2AR promotes Cbl-b-mediated Notch1 degradation *via* STS-1

**Figure 3:**
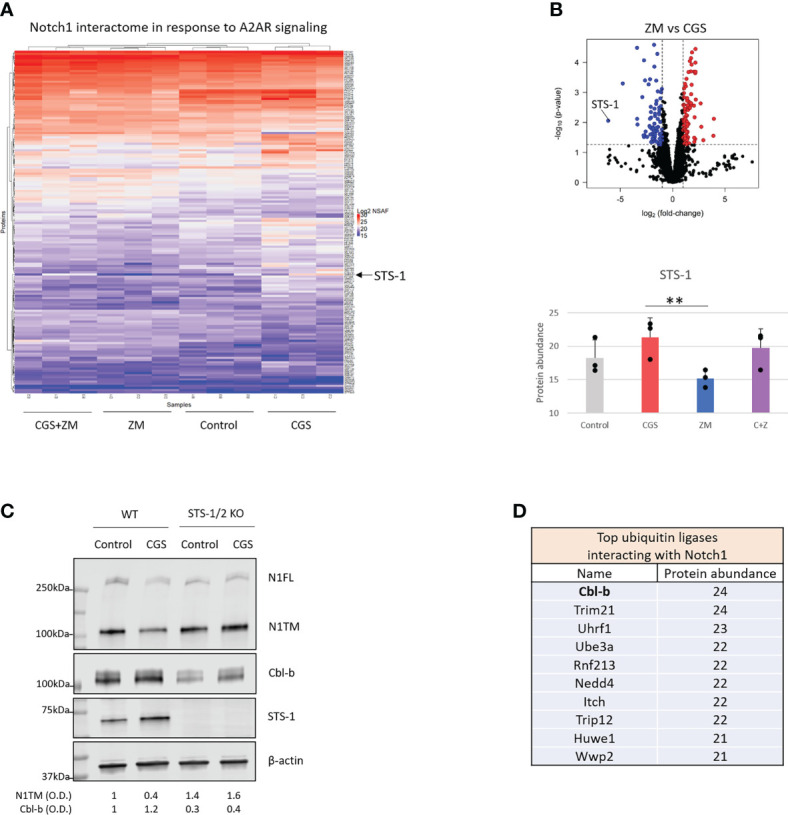
A2AR promotes Cbl-b-mediated Notch1 degradation *via* STS-1. Mass spectrometry analysis was carried out in lysates where Notch1 was immunoprecipitated from primary mouse CD8+ T-cells stimulated with anti-CD3/CD28 and treated with vehicle (control, DMSO) or 1µM ZM-241385 (ZM) or 1µM CGS-21680 (CGS) or ZM+CGS, as indicated in the figure. **(A)** Heat map of mass spectrometry analysis of Notch1 interactome. Uniprot IDs of the detected proteins are indicated on the right side of the heat map. Protein abundance is expressed as NSAF log2 fold-change on a color scale from blue to red where blue indicates the lowest and red the highest value, respectively. **(B)** Volcano plot of proteins identified in the mass spectrometry in ZM-treated *vs*. CGS-treated CD8+ T-cells. The X axis indicates fold change (log2) and Y axis the p-value (-log10). The dotted lines indicate the cut-off separating proteins with p<0.05 (blue and red dots) from non-significant ones (black dots). Proteins that are interacting the least with Notch1 are shifted to the left of the plot whereas the ones interacting the most are shifted to the right. The bottom graph shows the interaction between Notch1 and STS-1 (protein abundance) from the mass spectrometry analysis. **(C)** Protein levels of Notch1, STS-1 and Cbl-b in primary activated CD8+ T-cells isolated from C57BL6 (WT) or STS-1/2 -/- (STS-1/2 KO) mice and treated with vehicle (control, DMSO) or 1µM CGS. Densitometry (O.D.) results for N1TM and Cbl-b normalized by β-actin are shown below the panel. **(D)** Top 10 ubiquitin ligases interacting with Notch1 ordered by the most to the least interacting based on mass spectrometry results (protein abundance). The graphs show averages ± standard deviation from three independent experiments. **p<0.01, two tailed T-test with equal variance.

To determine how Notch1 degradation is regulated by A2AR, we analyzed the protein interactome of Notch1 in activated primary CD8+ T-cells treated with CGS or ZM (**Figure 3A**). From the mass spectrometry analysis STS-1 appeared as the Notch1-interacting protein that was changed the most by the treatments and more importantly, in opposite directions: CGS increased Notch1 interaction with STS-1, whereas ZM decreased this interaction (**Figures 3A, B**). This finding was of particular interest because STS-1 is a Tyr-phosphatase that acts as an important negative regulator of T-cell activation, but has never been linked with immunosuppression *via* adenosine and A2AR. To test whether STS-1 may control Notch1 degradation in response to A2AR activation, we detected Notch1 in STS-1/2 KO CD8+ T-cells treated with CGS. In line with our hypothesis, we found that Notch1 was not downregulated in STS-1/2 KO T-cells in response to CGS treatment (**Figure 3C**), indicating that lack of STS-1 prevents A2AR-induced Notch1 degradation. We next asked how STS-1 mediates the degradation of Notch1. Since we found that A2AR modulates the ubiquitination and degradation of Notch1 (**Figure 1**), we wanted to test whether STS-1 regulates Notch1 in CD8+ T-cells through a ubiquitin ligase. STS-1 is known to interact with ubiquitin ligases of the Cbl-family proteins through its SH3 domain and contribute to the regulation of target proteins (30–32). We analyzed the interactome of Notch1 in activated primary CD8+ T-cells using mass spectrometry (**Figure 3D**) and found that the Cbl-family protein, Cbl-b, was among the top ubiquitin ligases that interact the most with Notch1. Therefore, we asked whether STS-1 may promote Cbl-b and, in turn, Notch1 degradation. There is evidence of Cbl-b protein stability being positively regulated by Tyr-phosphatases (48) and, consistently, we found that Cbl-b was downregulated in STS-1/2 KO T-cells (**Figure 3C**), suggesting that STS-1 may stabilize Cbl-b and in turn, promote Cbl-b-mediated Notch1 degradation.

**Figure 4:**
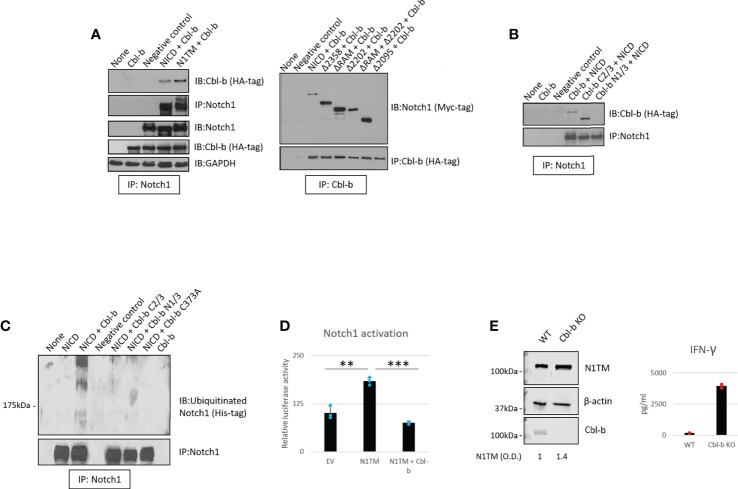
Cbl-b ubiquitinates and degrades Notch1. **(A, B)** Notch1 or Cbl-b co-immunoprecipitation in protein lysates from 293T cells were transfected with various Myc-tagged Notch1 and/or HA-tagged Cbl-b constructs, as indicated. **(C)** Ubiquitinated Notch1 protein levels detected upon immunoprecipitation of Notch1 in 293T cells co-transfected with a His-tagged NICD construct and various HA-tagged Cbl-b constructs, as indicated. **(D)** Notch1 activation in 293T cells measured using the Hes-luciferase reporter gene system. **(E)** Protein levels of Notch1 and IFN-gamma production in primary activated CD8+ T-cells isolated from C57BL6 (WT) or Cbl-b -/- (Cbl-b KO). Densitometry (O.D.) results for N1TM normalized by β-actin are shown below the panel. The graphs show averages ± standard deviation from three independent experiments. **p < 0.01, ***p < 0.001 two tailed T-test with equal variance. Negative control refers to samples immunoprecipitated using rabbit IgG instead of anti-Notch1/HA-tag/Cbl-b antibody. None, non-transfected cells. EV, empty vector.

We then asked whether Cbl-b mediates the regulation of Notch1. To confirm the interaction between Notch1 and Cbl-b and map the interacting sites, we set up a series of immunoprecipitation assays using 293T cells transfected with various Myc-tagged Notch1 and HA-tagged Cbl-b constructs (**Figures 4A, B**). We tested whether Cbl-b could associate with transmembrane Notch1 (N1TM) as well as the cleaved, transcriptionally active form of Notch1 (NICD). Cbl-b was pulled down with both Notch constructs, however, N1TM always showed slightly higher affinity for Cbl-b than NICD at similar expression levels (**Figure 4A**). Next, we attempted to identify the regions indispensable for Notch1 - Cbl-b interaction. First, 293T cells were transfected with a series of Notch1 constructs in which an increasing amount of the C-terminal region was deleted, along with the HA-tagged full length Cbl-b construct. Among the various deletion mutants used, only Δ2095, a construct that has part of the transcriptional activation domain (TAD) deleted, did not bind Cbl-b (**Figure 4A**). Reciprocal experiments using HA-tagged Cbl-b deletion mutants revealed that the C-terminal region of Cbl-b is important for binding Notch1 (**Figure 4B**). Deletion of the protein kinase binding domain (TKB) at the N-terminus of Cbl-b in mutant Cbl-b C2/3, did not impair the binding with Notch1, whereas a mutant consisting of TKB only (Cbl-b N1/3) was unable to pull down Notch1 (**Figure 4B**). Lastly, we confirmed that Cbl-b could be immunoprecipitated with Notch1 and STS-1 in primary CD8+ T-cells (**Supplementary Figure 3**). These data showed that Cbl-b and Notch1 associate with each other and a region containing part of the TAD domain of NICD and the C-terminal region of Cbl-b are important for this interaction. This is consistent with the observations that other Notch-targeting ubiquitin ligases bind to NICD (24) and Cbl-b interacts with its targets and SH3-domain containing proteins, like STS-1, through its C-terminal region (41, 49).

As Notch1 ubiquitination is increased in CD8+ T-cells in response to A2AR activation (**Figure 1**), we asked if Cbl-b regulates Notch1 signaling by directly ubiquitinating Notch1 protein. To do this, 293T cells were transfected with a His-tagged ubiquitin construct along with Notch1 and Cbl-b constructs (**Figure 4C**). We detected the presence of ubiquitinated Notch1 only in the cells that were co-transfected with Notch1 and full-length Cbl-b. N- and C-terminal deletion mutants, as well as a point mutant of Cbl-b (C373A) which is deficient in its E3 ligase activity, were unable to ubiquitinate Notch1 (**Figure 4C**).

To determine whether Cbl-b-mediated Notch1 ubiquitination leads to Notch1 signaling downregulation, we used a reporter gene assay based on a reporter construct in which the luciferase gene is placed downstream of a target of Notch1, the *Hes* promoter (50). Reporter gene activity was reduced two- to three-fold when Notch1 and Cbl-b were co-transfected in 293T cells, compared to Notch1 alone (**Figure 4D**), indicating that Cbl-b negatively regulated Notch1 signaling. In agreement with these results, we found that Notch1 was upregulated in Cbl-b KO primary CD8+ T-cells compared to WT cells (**Figure 4E**) and led to increased IFN-gamma production, suggesting that lack of Cbl-b rescues Notch1 from degradation and restores the transcription of its target genes. Taken together, these results indicate that Cbl-b ubiquitinates and degrades Notch1 and lack of Cbl-b is sufficient to restore Notch1 protein levels and signaling. Our data supports a model in which A2AR controls, *via* STS-1, Cbl-b-mediated Notch1 degradation.

### Genetic KO and pharmacologic inhibition of Cbl-b rescue Notch1 and T-cell functions from A2AR-mediated immunosuppression

**Figure 5:**
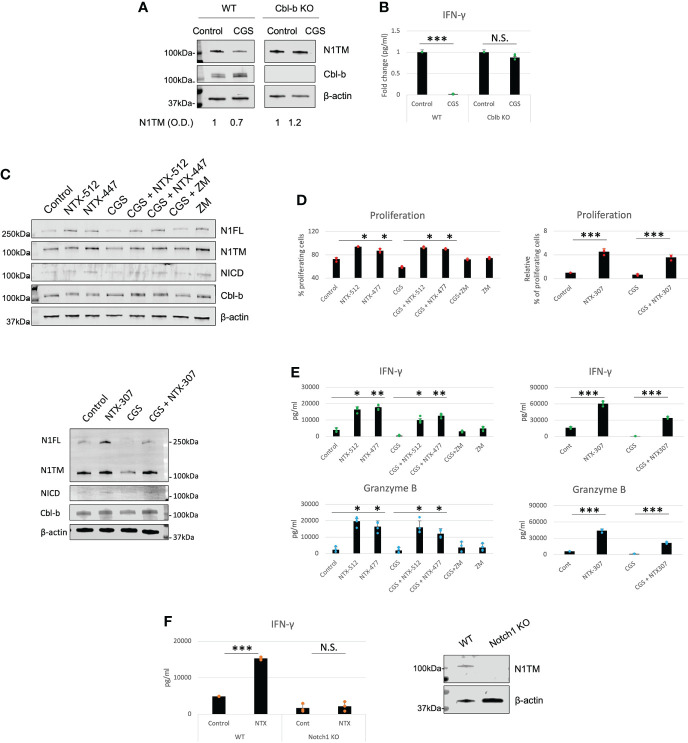
Genetic KO and pharmacologic inhibition of Cbl-b rescue Notch1 and T-cell functions from A2AR-mediated immunosuppression. **(A)** Protein levels of Notch1 and **(B)** IFN-gamma production in primary activated CD8+ T-cells isolated from C57BL6 (WT) or Cbl-b -/- (Cbl-b KO). Densitometry (O.D.) results for N1TM normalized by β-actin are shown below the panel. **(C)** Protein levels of Notch1 in primary activated CD8+ T-cells and treated with Cbl-b inhibitors (1µM NTX-512, NTX-447, and NTX-307) or 1µM ZM-241385 (ZM) or 1µM CGS-21680 (CGS) or combinations, as indicated in the figure. **(D)** Proliferation, **(E)** IFN-γ and Granzyme B in primary CD8+ T-cells treated as in **(C)**. **(F)** IFN-γ production in unmodified or CRISPR-Cas9 Notch1 KO primary activated CD8+ T-cells untreated (control) or treated with 1µM NTX-307. The panel in **(F)** shows Notch1 protein levels in unmodified *vs*. Notch1 KO cells. The graphs show averages ± standard deviation from three independent experiments. *p < 0.05, **p < 0.01, ***p < 0.001. two tailed T-test with equal variance. N.S, non-significant.

Our data place Cbl-b at the core of an immunosuppressive pathway connecting A2AR, Notch1 and T-cell functions. This is also consistent with previous work that showed that Cbl-b is a key negative regulator of T-cell activation (28, 29). Therefore, we asked if genetic KO or pharmacological inhibition of Cbl-b could be a strategy to enhance T-cell functions and counteract tumor-induced immunosuppression by promoting Notch1. We treated CD8+ T-cells from Cbl-b KO mice with CGS and detected Notch1 level and IFN-gamma (**Figures 5A, B**). In agreement with our hypothesis, we found that CGS did not reduce Notch1 and INF-gamma in Cbl-b KO CD8+ T-cells (**Figures 5A, B**), confirming that lack of Cbl-b prevents Notch1 downregulation and, in turn, promotes T-cell function. Considering these results, we asked if pharmacologic inhibition of Cbl-b could recapitulate what we observed in Cbl-b KO CD8+ T-cells. To accomplish this, we tested the effect of novel small molecule investigational compounds (NTX, Nimbus Therapeutics), designed to inhibit Cbl-b, in primary CD8+ T-cells. NTX compounds were designed using a structure-guided approach to inhibit Cbl-b enzymatic activity. This structure-based approach ensures exquisite selectivity towards Cbl-b and c-Cbl, another member of the Cbl-family of ubiquitin ligases. In this study, we selected three candidate compounds, NTX-512, NTX-447 and NTX-307, based on potency. The potency of the compounds to inhibit Cbl-b was assessed by time-resolved measurement of fluorescence with fluorescence resonance energy transfer technology (TR-FRET) (**Supplementary Figure 4**). Consistent with our data in Cbl-b KO T-cells, we found that Cbl-b inhibitors, NTX-512, NTX-447 and NTX-307, rescue both full length and cleaved forms of Notch1 from CGS-mediated downregulation, suggesting that Cbl-b inhibition prevents Notch1 downregulation (**Figure 5C**). The effect of Cbl-b inhibitors on Notch1 resulted in rescue of proliferation, IFN-gamma and Granzyme B (GNZB) production from CGS-mediated suppression and increased proliferation and production of cytokines compared to vehicle-treated CD8+ T-cells (**Figures 5D, E**). The compounds showed dose-dependent effect on proliferation and cytokines production and remarkable potency as indicated by low EC50s both as single agents and against CGS (**Supplementary Figures 5A, B**). Importantly, the compounds did not increase the production of IFN-gamma in unstimulated CD8+ T-cells, indicating that they only boost the function of antigen-stimulated CD8+ T-cells (**Supplementary Figure 5C**). Finally, Cbl-b inhibitors failed to increase IFN-gamma production in somatic (CRISPR/Cas9-mediated) Notch1 KO primary CD8+ T-cells, suggesting that the Cbl-b inhibitor effect on T-cell function is Notch1-dependent (**Figure 5F**). These results strongly indicate that Cbl-b inhibition boosts Notch1 and T-cell functions and renders CD8+ T-cells resistant to A2AR-mediated immunosuppression.

### Cbl-b inhibitors enhance CD8+ T-cells anti-tumor responses as single-agent and combinatorial immunotherapy with immune-checkpoint inhibitors

**Figure 6:**
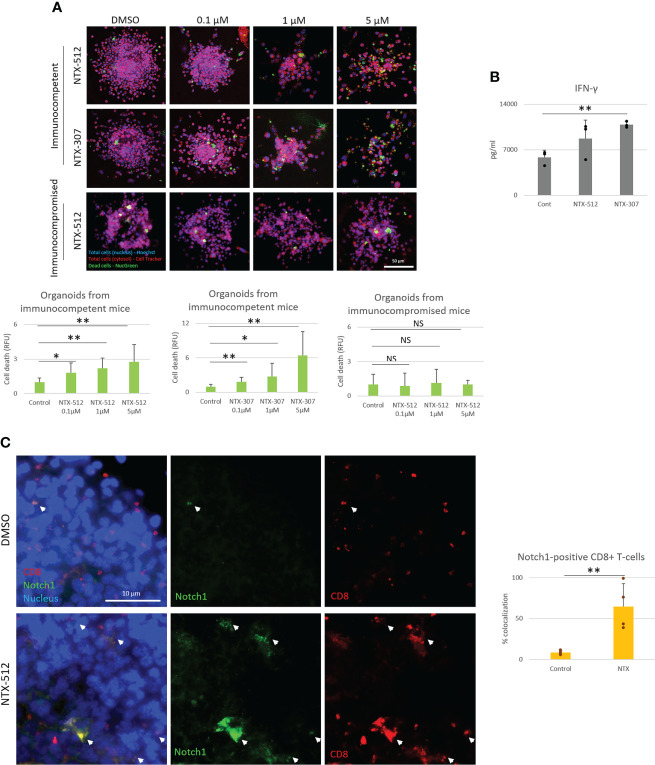
Cbl-b inhibitors enhance Notch1-positive CD8+ T-cells anti-tumor responses. Organoids were derived from a syngeneic TNBC mouse model, C0321, in FVB mice or immunocompromised athymic Nu/Nu mice and treated with vehicle (control, DMSO) 0.1, 1 and 5µM of NTX-512 or NTX-307, as indicated in the figure. **(A)** Cancer cell death, **(B)** production of IFN-gamma in organoid cultures and **(C)** colocalization of Notch1 and CD8+ T-cells in organoids indicated with white arrows in the panel and in the plot. Panel **(C)** shows threshold-adjusted images to better highlight Notch1 staining in CD8+ T-cells, whereas the original images are presented in **Supplementary Figure 7B**. Scale bars length is indicated above each bar (µm). The graphs show averages ± standard deviation from ≥ 10 organoids from at least three independent experiments. *p < 0.05, **p < 0.01, two tailed T-test with equal variance. NS, non-significant.

Since Cbl-b inhibition enhances T-cell function and resistance to A2AR-mediated-immunosuppression, we next wanted to test whether the effect of Cbl-b inhibition could translate into increased anti-tumor T-cell responses. *In vivo* experiments were not attempted as the pharmacokinetics of the novel inhibitors is still under investigation. Therefore, to answer this question, we treated TNBC C0321 tumor-derived organoids with different concentrations of Cbl-b inhibitors and analyzed several readouts for anti-tumor activity. We found that Cbl-b inhibitors significantly induced cell death in organoids in a dose-dependent manner (**Figure 6A**). This effect was absent in organoids derived from immunocompromised atymic Nu/Nu mice, suggesting that the anti-cancer activity of the compounds is immunologically mediated (**Figure 6A**). Concomitantly, we found that Cbl-b inhibitors-treatment increased the production of IFN-gamma in infiltrating CD8+ T-cells and in organoids cultures (**Figure 6B**, **Supplementary Figure 6**), a sign of increased CD8+ T-cell activation. Lastly, we labeled CD8+ T-cells and Notch1 in organoids and observed a significant infiltration of Notch1-positive CD8+ T-cells in organoids treated with Cbl-b inhibitors compare to control organoids (**Figure 6C**). These Notch1-positive T-cells were surrounding cancer cells, possibly establishing immunological synapses for cancer cell killing. We also confirmed that Cbl-b inhibitors did not affect Notch1 in cancer cell lines, including C0321, and cancer cells in organoids (**Supplementary Figure 7**). Overall, our results indicate that Cbl-b inhibition enhances CD8+ T-cell anti-cancer responses and show potential as single-agent cancer immunotherapy.

**Figure 7:**
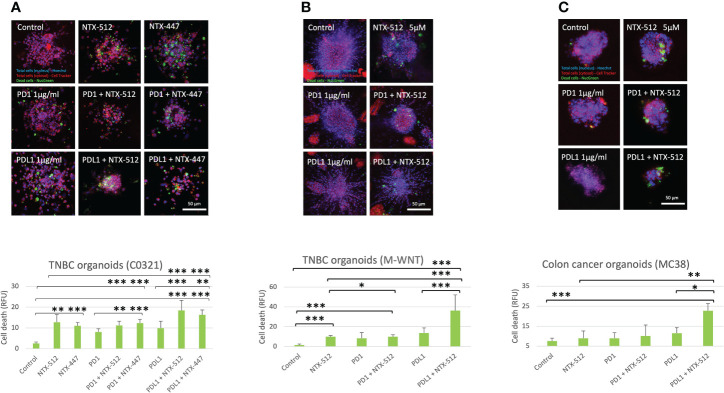
Cbl-b inhibitors enhance anti-PD1/PDL1 efficacy in tumor-derived organoids. Organoids were obtained from syngeneic TNBC, **(A)** C0321, **(B)** M-WNT, and colon cancer, **(C)** MC38, models and treated with vehicle (control, DMSO) or 5µM of NTX-512 or NTX-477, alone or in combination with 1µg/ml anti-PD1 or 1µg/ml anti-PDL1. The plots show cancer cell death in organoids. Scale bars length is indicated above each bar (µm). The graphs show averages ± standard deviation from ≥10 organoids from independent experiments. *p < 0.05, **p < 0.01, ***p < 0.001, one-way ANOVA. NS, non-significant.

Immune-checkpoint inhibitor therapy is FDA-approved for the treatment of certain tumors, including TNBC, but only a limited number of patients benefit from it (51). Therefore, we tested if Cbl-b inhibitors could enhance the efficacy of anti-PD1/PDL1 immunotherapy. We treated organoids derived from two genetically distinct pre-clinical models of TNBC, C0321 and M-WNT (37–39), and a model of colon cancer, MC38, with Cbl-b inhibitors (**Figure 7**). We choose TNBC and colon cancer models since both tumors are known to have an immunosuppressive microenvironment (52, 53). The compounds were effective in inducing cell death in organoids both as single agents and in combination with anti-PD1/anti-PDL1 (**Figure 7**). Interestingly, a marked enhancement of anti-tumor activity was seen when Cbl-b inhibitors and anti-PDL1 were used in combination in all models (**Figure 7**), thus highlighting the possibility that these classes of agents could be used for combinatorial immunotherapy. Our results show that Cbl-b inhibitors, by inducing Notch1-dependent CD8+ T-cells responses, have a promising potential as anti-cancer single-agents and as combinatorial immunotherapy with checkpoint inhibitors. Overall, Cbl-b inhibition represents a new immunotherapeutic strategy that could be exploited to sensitize tumors to anti-cancer T-cell responses and treat tumors that are refractory to immunotherapy.

## Discussion

Tumor-induced immunosuppression is a critical feature of cancer that allows evasion from the immune system (1). This is a major challenge for designing effective cancer immunotherapies that circumvent immunosuppression and provide significant response rates.

**Figure 8:**
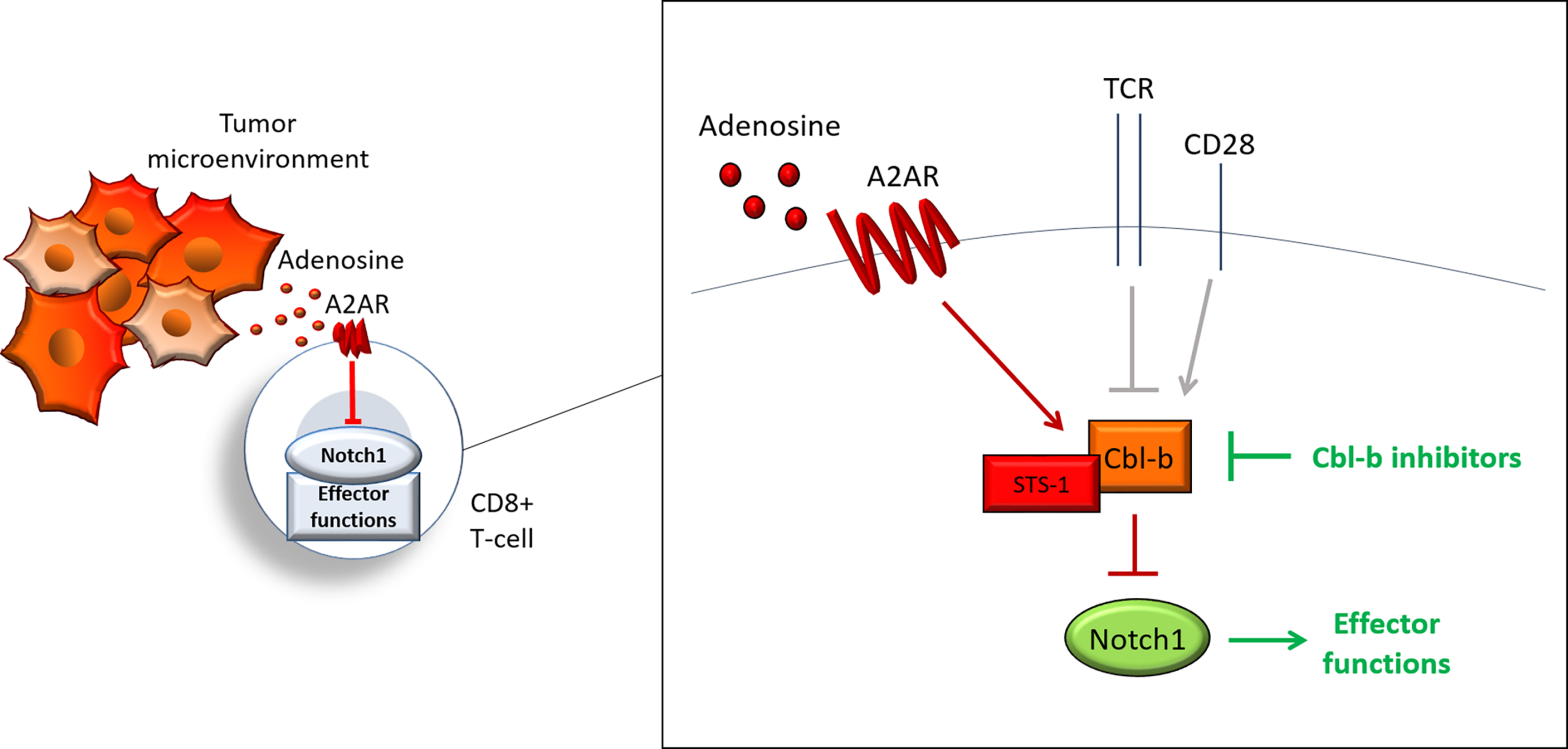
Cbl-b-Notch1 axis regulation. Adenosine induces immunosuppression in CD8+ T-cells in the tumor microenvironment by activating the adenosine A2A receptor (A2AR), thus causing downregulation of Notch1 and suppression of effector functions. A2AR controls Notch1 levels by regulating the ubiquitin ligase Casitas B-lineage lymphoma b (Cbl-b), which mediates the ubiquitination and degradation of Notch1. A2AR promotes Cbl-b *via* Suppressor of T-cell receptor signaling 1 (STS-1) Tyr-phosphatase, possibly through Tyr-dephosphorylation of Cbl-b. A2AR, together with other receptors, including T-cell receptor (TCR) and co-stimulation receptor CD28, may control Notch1 levels in T-cells and, in turn, effector functions by modulating Cbl-b. This model places Cbl-b at the core of a regulatory axis, which, by integrating positive and negative signals from different receptors, determines the fate of Notch1 and effector functions. Pharmacological inhibition of Cbl-b blocks this axis and reactivates Notch1 and effector functions.

Our work describes a new regulatory pathway that is critical for tumor-induced immunosuppression in CD8+ T-cells and demonstrates that targeting this pathway is a promising strategy to overcome immunosuppression and enhance anti-cancer responses. We showed that activation of A2AR by adenosine, promotes Cbl-b-mediated Notch1 ubiquitination and degradation. STS-1 Tyr-phosphatase associates with Notch1 in response to A2AR activation and coordinates Cbl-b-mediated Notch1 degradation (**Figure 8**). Genetic KO of Cbl-b increases Notch1 levels and signaling. Similarly, pharmacological inhibition of Cbl-b results in increased Notch1, T-cell functions, anti-cancer response and resistance to immunosuppression in TNBC and colon cancer models. We also provided evidence illustrating that combination of Cbl-b inhibition and immune-checkpoint inhibition has enhanced efficacy in the same models.

By identifying the Cbl-b-Notch1 axis in CD8+ T-cells, we showed for the first time a direct link between Cbl-b and adenosine-mediated immunosuppression, and placed Cbl-b and STS-1 at the center of Notch1 regulation in CD8+ T-cells. Our findings, suggest that the Cbl-b-Notch1 axis could represent a new functional immune-checkpoint that dampens T-cell responses in the tumor microenvironment and that blockade of this pathway may be a key strategy to overcome tumor-induced immunosuppression. Future work will need to determine the significance of this pathway in the tumor microenvironment as well as under physiological conditions, and its potential applications for cancer immunotherapy.

Our work supports a model in which both immunosuppressive and activating signals may converge onto the Cbl-b-Notch1 axis and may be translated by Notch1 into transcriptional (and potentially non-transcriptional) signals to regulate T-cell activation (**Figure 8**). In addition, Cbl-b may mediate a constitutive degradative pathway that is switched on and off depending on whether the cell requires more or less Notch1. In T-cells Notch1 is activated in a ligand-independent manner through endocytosis (10). It is possible that Cbl-b degradative pathway could be part of the ligand-independent endocytic pathway of Notch1 in T-cells, since endocytic regulation of Notch can either lead to activation or degradation (10, 21, 23). In this endocytic pathway, Notch1 may be directed to degradation by Cbl-b-mediated ubiquitination, whereas other signals may instead direct Notch1 to activation, as observed in other systems (24, 26). This degradation pathway may be controlled in response to extracellular stimuli, including TCR activation and A2AR signaling, which are both known to regulate the level of Notch1 in T-cells (7, 10, 12, 13). For example, upon T-cell activation, signaling downstream of TCR may inhibit Cbl-b to elevate Notch1 and effector functions. Later, to avoid over-activation and exhaustion of T-cells, co-stimulatory signals (e.g. CD28) or immunosuppressive ones (e.g. A2AR) may promotes Cbl-b-mediated degradation of Notch1 and downregulation of effector functions (**Figure 8**). This would explain at least in part how Cbl-b controls the threshold for T-cell activation (28, 29).

Previous work and our data, suggest that Cbl-b-mediated degradation may be regulated through phosphorylation. Cbl-b is negatively regulated by Tyr-phosphorylation by SHP-1 or Ser/Thr-phosphorylation by PKC-theta in response to CD28 co-stimulation, but dephosphorylated and promoted in response to TCR stimulation (48, 54, 55). Accordingly, our results suggest that Cbl-b may be positively regulated by STS-1, possibly through Tyr-dephosphorylation, and promoted upon A2AR activation. It is very likely that the phosphorylation of different residues or a different phosphorylation status of Cbl-b may increase or decrease Cbl-b function and/or stability. If Cbl-b is controlled by phosphorylation, it is also possible that a Tyr-kinase antagonizes STS-1 function, by phosphorylating and inhibiting Cbl-b. Overall, our work supports the idea that antagonistic signals from TCR and other receptors, like A2AR, control Cbl-b and, in turn, effector functions *via* Notch1, thus regulating the threshold of T-cell activation. This regulation, including the signals it responds to, the function of STS-1 and the possible involvement of Cbl-b in the endocytic trafficking of Notch1, warrant further investigation.

**Figure 9:**
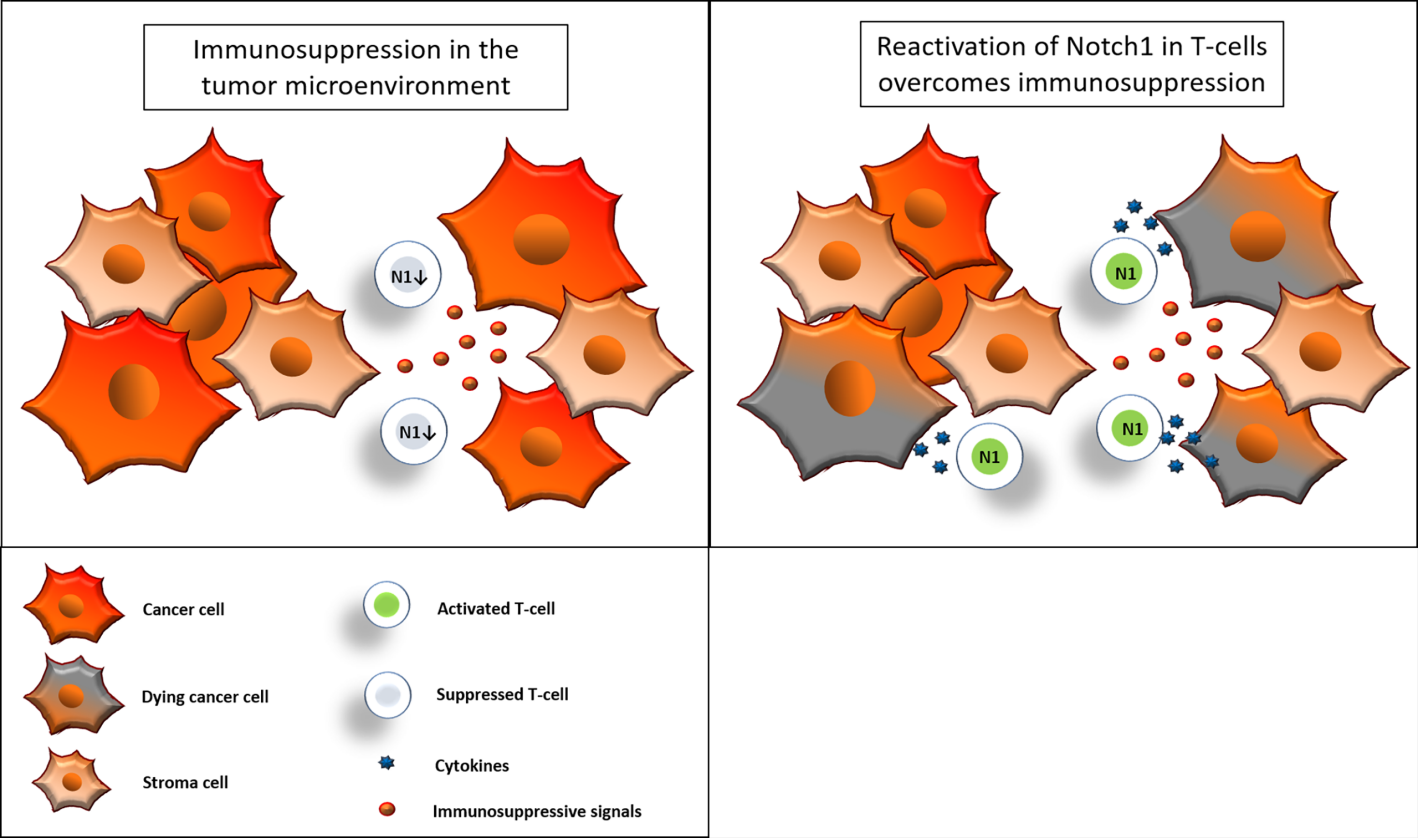
Reactivation of Notch1 in T-cells overcomes immunosuppression. Immunosuppressive signals induce suppression in CD8+ T-cells in the tumor microenvironment. Some of these signals, including adenosine, may suppress CD8+ T-cell effector functions by downregulation of Notch1. Reactivation of Notch1, with Cbl-b inhibitors or other strategies, may tune CD8+ T-cells against immunosuppression and enhance effector functions, ultimately promoting anti-cancer responses in the tumor microenvironment.

Despite the successful application of immunotherapy, response rates remain limited especially for immunosuppressive solid tumors (51). New immunotherapies that are less sensitive to different forms of tumor-induced immunosuppression could greatly increase response rates among cancer patients. Our work, together with previous studies (7, 15), supports the idea that therapies that reactivate Notch1 in T-cells could be used to tune T-cells against tumor-induced immunosuppression and enhance anti-cancer immune responses (**Figure 9**). Specifically, we described a new pathway that is amenable to pharmacological targeting and has promising selectivity for Notch1 in T-cells versus cancer cells, a feature that is very important for Notch-targeted therapies (16). By reactivating Notch1 in T-cells we aim to lower the threshold of T-cell activation to make tumor-suppressed T-cells more responsive to tumor antigen recognition when infiltrating the tumor microenvironment. Consistently, we observed that reactivation of Notch1 increased/restored effector functions and primed T-cells to attack cancer cells in tumor-derived organoids. Our results in organoids from TNBC and colon cancer, two tumor types which can suppress immune responses (52, 53), suggest that Notch1 reactivation *via* Cbl-b inhibition could be a promising strategy to sensitize “cold” immunosuppressive tumors to cancer immunotherapy. Another promising application of this strategy, could be adoptive T-cell therapies. Resistance to immunosuppression plays a critical role in these therapies and both Notch1 expression and Cbl-b deletion were found to enhance the efficacy of adoptive T-cell transfer and CAR-T cell therapy (15, 36). Our work presents a new class of candidate immunotherapeutic compounds, Cbl-b inhibitors, that enhance anti-cancer T-cell responses and resistance to tumor-induced immunosuppression. We show that Cbl-b inhibitors are effective as single-agents or in combination with anti-PDL1/PD1 in organoids derived from pre-clinical cancer models, thus these compounds also have the potential to enhance responses to other immunotherapies. The pharmacokinetic and bioavailability of Cbl-b inhibitors is currently under investigation and future work will focus on evaluating the compounds anti-tumor activity *in vivo* in pre-clinical models and their potential clinical development.

Our work described for the first time a critical immunosuppressive pathway linking A2AR, Cbl-b and Notch1, which could represent a new functional immune-checkpoint that modulates T-cell responses in the tumor microenvironment. We showed that promoting Notch1 signaling by blocking Cbl-b-mediated degradation results in a robust increase in anti-cancer T-cell responses and resistance to immunosuppression. Our findings provide evidence that targeting Cbl-b-Notch1 axis represents a promising novel immunotherapeutic strategy to boost anti-cancer T-cell responses and overcome tumor-induced immunosuppression.

## Data availability statement

The datasets presented in this study can be found in online repositories. The names of the repository/repositories and accession number(s) can be found in the article/**Supplementary Material**.

## Ethics statement

The animal study was reviewed and approved by the Institutional Animal Care and Use Committee (IACUC) at LSU Health Sciences Center - New Orleans.

## Author contributions

GM, ZH, LM, CL, BO, and SM contributed to the conception and design of the study. GM, ZH, FC, SL, DC, AC, JA, DU, FH, SI, AB, NC, KX, and SM performed experiments and/or provided key materials. GM wrote the first draft of the manuscript. All authors contributed to manuscript revision, read, and approved the submitted version.

## Funding

This work was supported in part by by the U.S. Department of Defense (DoD; PR200633; Monticone G.) and the National Institute of Health (NIH; P20CA233374; Miele L. and Ochoa A.). We thank Nimbus Therapeutics for providing the Cbl-b inhibitors; Dr. Jeffrey Chiang for providing Cbl-b KO T-cells; Dr. Samuel Mackintosh, his team and the IDeA proteomics program for the mass spectrometry analysis; Dr. Stephen D. Hursting for providing M-WNT cells; Drs. Maira Di Tano, Cristina Melero, Francesca Carrieri, Yue Qu and Ramesh Thylur Puttalingaiah for providing insightful discussions; Dr. Augusto Ochoa, Dr. Krzysztof Reiss and their research groups for providing intellectual and technical assistance.

## Conflict of interest

Authors FC, SLt, DC, and CL were employed by company Nimbus Theraputics.

The remaining authors declare that the research was conducted in the absence of any commercial or financial relationships that could be construed as a potential conflict of interest.

## Publisher’s note

All claims expressed in this article are solely those of the authors and do not necessarily represent those of their affiliated organizations, or those of the publisher, the editors and the reviewers. Any product that may be evaluated in this article, or claim that may be made by its manufacturer, is not guaranteed or endorsed by the publisher.

## Supplementary material

Supplementary Figure 1 A2AR activation increases Notch1 degradation. Notch1 protein levels in primary activated CD8+ T-cells treated with the protein synthesis inhibitor Cycloheximide (CHX) and vehicle (DMSO, control) or 1µM CGS-21680 (CGS), over time.

Supplementary Figure 2 CD8+ T-cells infiltration and organoid growth. **(A)** CD8+ T-cells in TNBC C0321 organoids, from immunocompetent FVB mice, treated with vehicle (DMSO) or 1µM ZM-241385 (ZM). **(B)** Growth of organoids, from immunocompromised athymic Nu/Nu mice, treated with vehicle (DMSO) or 1µM ZM-241385 (ZM). Scale bar length is indicated above the bar (µm). The graphs show averages ± standard deviation from ≥4 independent experiments. *p<0.05, two tailed T-test with equal variance. NS, non-significant.

Supplementary Figure 3 Co-immunoprecipitation of Cbl-b, Notch1 and STS-1. Immunoprecipitation of Cbl-b and co-detection of Notch1 and STS-1 in primary activated CD8+ T-cells. Negative control refers to samples immunoprecipitated using beads but not antibody.

Supplementary Figure 4 Potency studies of NTX compounds. The potency of NTX compounds to inhibit Cbl-b was assessed by time-resolved measurement of fluorescence with fluorescence resonance energy transfer technology (TR-FRET). The figure shows dose-response curves and IC50s calculated using a 4-parameter dose-response equation. The X axis of the graphs show concentrations (µM) in log10. The Y axis show the responses (Cbl-b inhibition) expressed as percentages. Data was normalized using high and low assay controls: % Inhibition =100-(100*[(high control) - unknown)/(high control - low control)].

Supplementary Figure 5 Cbl-b inhibitors show a dose-response effect in activated T-cells and no effect in unstimulated T-cells. Dose-response curves and EC50s of proliferation and production of IFN-γ and Granzyme B (GNZB) in primary activated CD8+ T-cells isolated treated with different concentrations of **(A)** NTX-512 alone or against **(B)** 1µM CGS-21680 (CGS). The X axis of the graphs show concentrations (µM) in log10. The Y axis show the responses (proliferation or cytokine production) expressed as percentages. For clarity, the highest response value was set to 100% and the other values were set accordingly. **(C)** Production of INF-γ in activated or unstimulated primary CD8+ T-cells and treated with 1µM NTX-512 or vehicle-treated (Control, DMSO). The graphs show averages ± standard deviation from three independent experiments. ***p<0.001. two tailed T-test with equal variance. N.S, non-significant.

Supplementary Figure 6 Cbl-b inhibitors increase the production of IFN-gamma in infiltrating CD8+ T-cells. Percentage of IFN-gamma+ CD8+ T-cells in organoids untreated (DMSO) or treated with NTX-512. IFN-gamma+ CD8+ T-cells were gated for live cells, CD45+ and CD3+.

Supplementary Figure 7 Cbl-b inhibitors do not modify Notch1 in cancer cells. **(A)** Protein levels of Notch1 in TNBC cancer cell lines, C0321, M-WNT, and colon cancer, MC38, treated with vehicle (control, DMSO) or 0.01, 0.1, 1 and 5µM of NTX-512. The graphs show the densitometry analysis of NICD normalized by β-actin. The graphs show averages ± standard deviation from three independent experiments. Two tailed T-test with equal variance identify no significant differences in NICD in the samples. **(B)** Notch1 and CD8+ T-cell staining in C0321 organoids treated with vehicle (control, DMSO) or NTX-512. White arrows indicate co-localization of Notch1 and CD8+ T-cells. This panel show the images presented in without threshold adjustment to show Notch1 staining in surrounding cancer and stroma cells in organoids.
