# Supplementary figures and images for: Targeting the Cbl-b-Notch1 axis as a novel immunotherapeutic strategy to boost CD8+ T-cell responses
Source: Front Immunol. 2022 Aug 26;13:987298. doi: 10.3389/fimmu.2022.987298 (PMC9459147; doi:10.3389/fimmu.2022.987298)

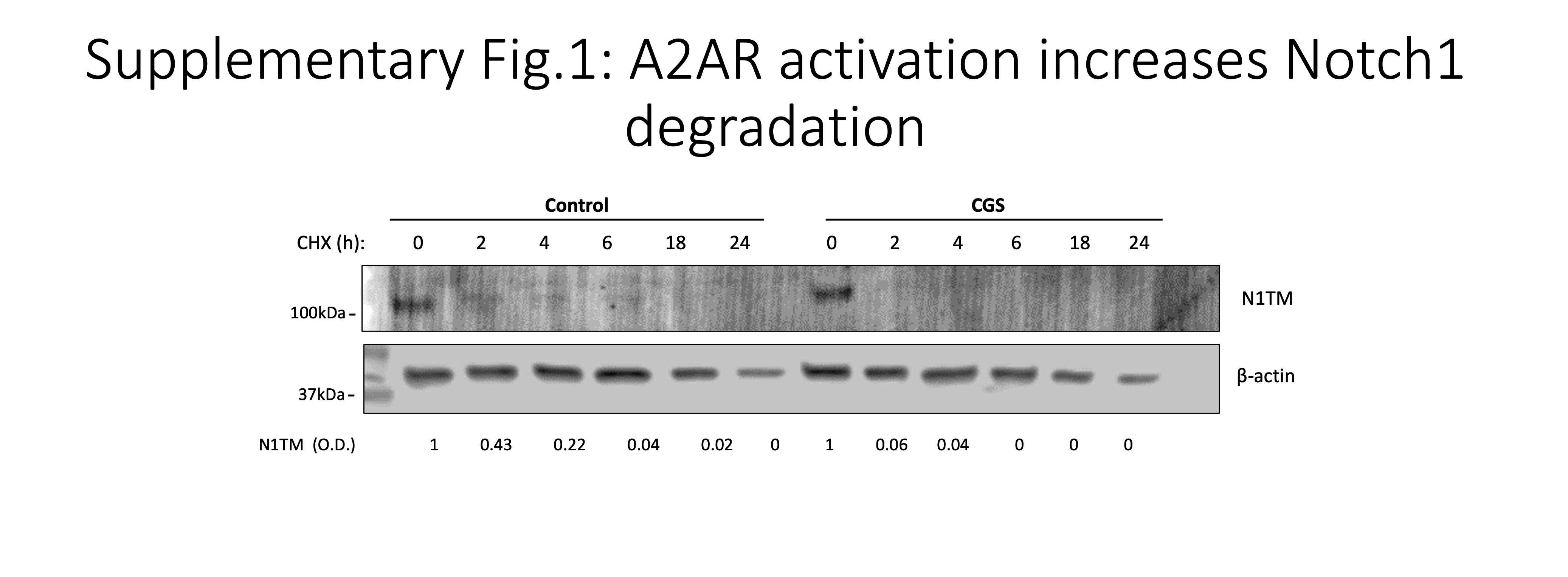

Supplement: Supplementary Figure 1 — A2AR activation increases Notch1 degradation. Notch1 protein levels in primary activated CD8+ T-cells treated with the protein synthesis inhibitor Cycloheximide (CHX) and vehicle (DMSO, control) or 1µM CGS-21680 (CGS), over time. [file Image_1.jpeg]

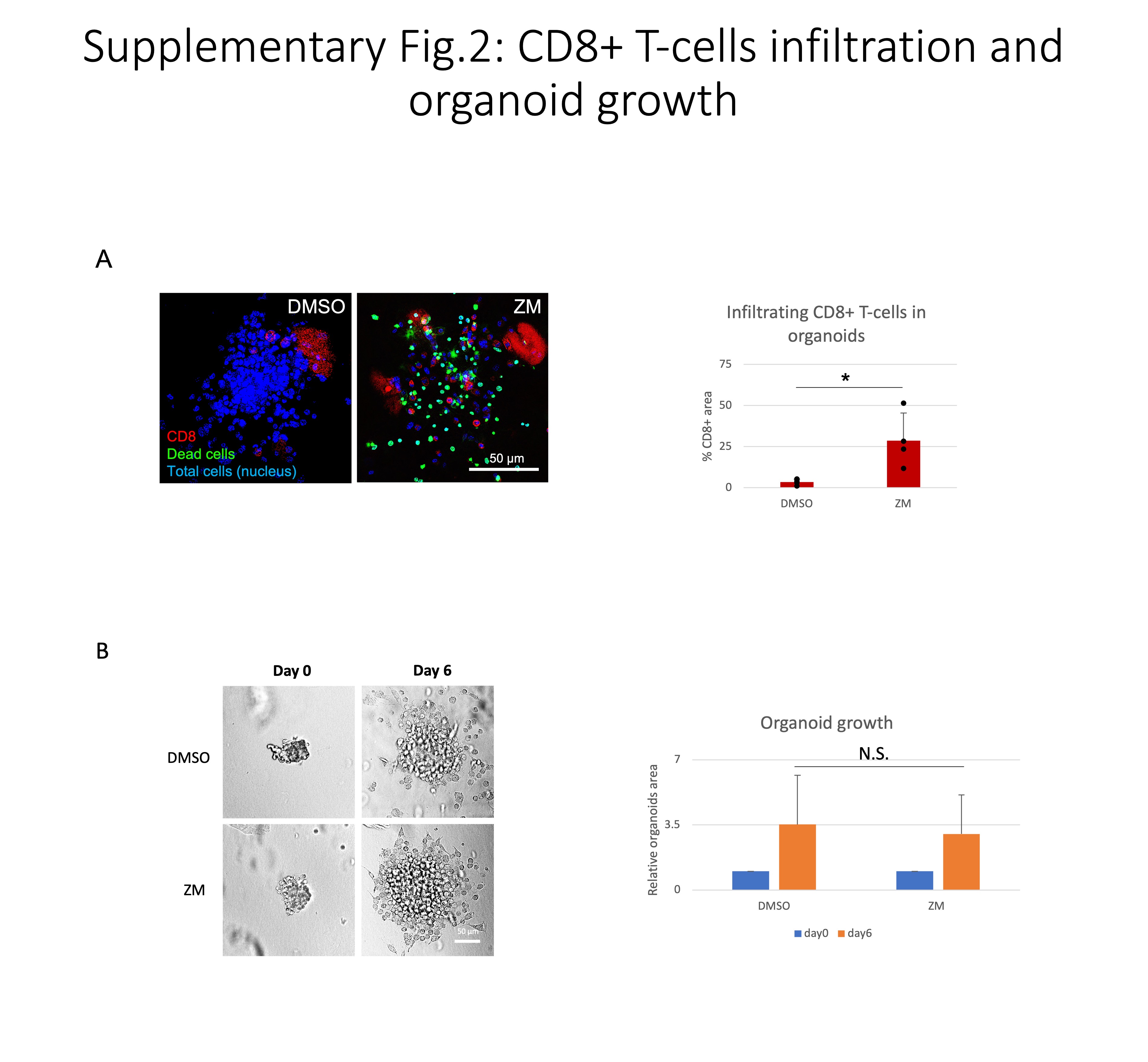

Supplement: Supplementary Figure 2 — CD8+ T-cells infiltration and organoid growth. (A) CD8+ T-cells in TNBC C0321 organoids, from immunocompetent FVB mice, treated with vehicle (DMSO) or 1µM ZM-241385 (ZM). (B) Growth of organoids, from immunocompromised athymic Nu/Nu mice, treated with vehicle (DMSO) or 1µM ZM-241385 (ZM). Scale bar length is indicated above the bar (µm). The graphs show averages ± standard deviation from ≥4 independent experiments. *p<0.05, two tailed T-test with equal variance. NS, non-significant. [file Image_2.jpeg]

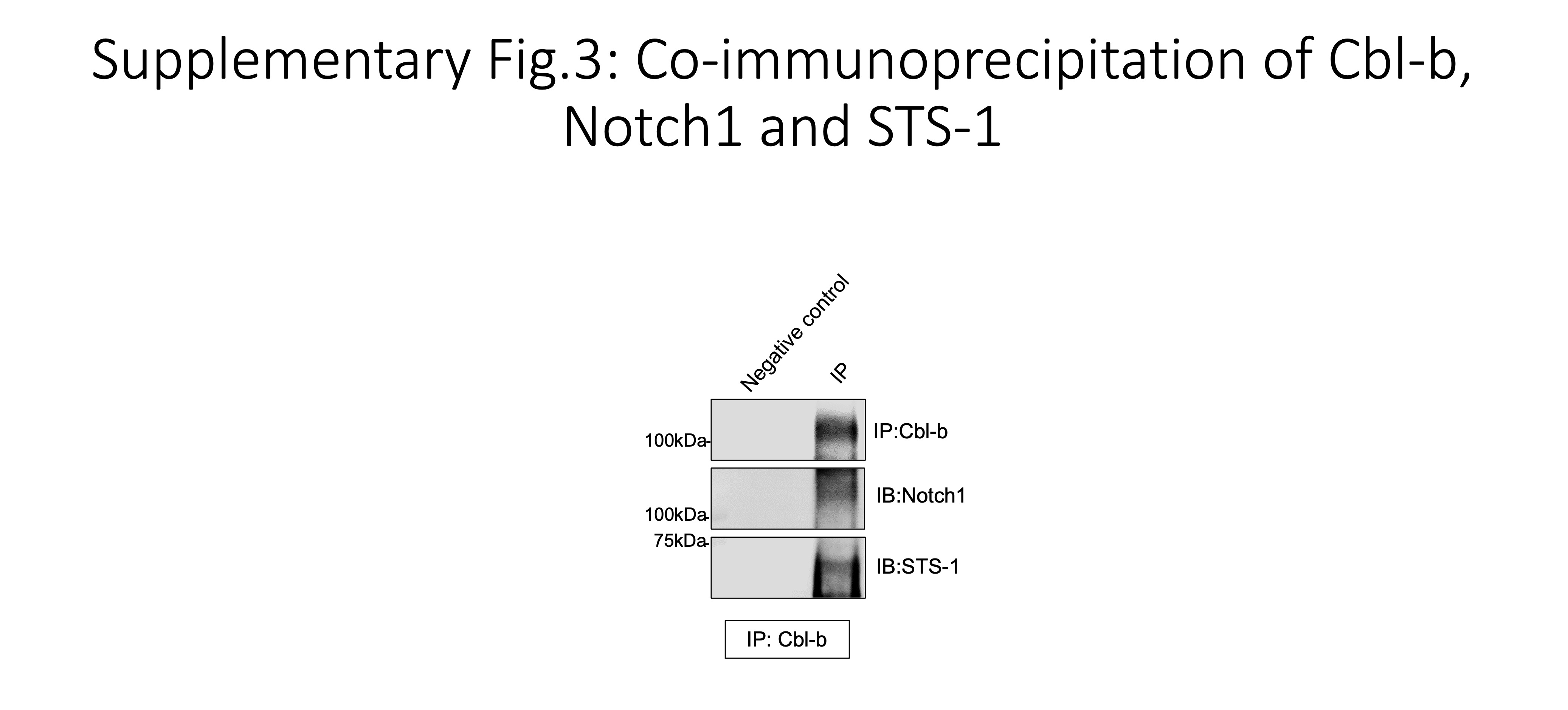

Supplement: Supplementary Figure 3 — Co-immunoprecipitation of Cbl-b, Notch1 and STS-1. Immunoprecipitation of Cbl-b and co-detection of Notch1 and STS-1 in primary activated CD8+ T-cells. Negative control refers to samples immunoprecipitated using beads but not antibody. [file Image_3.jpeg]

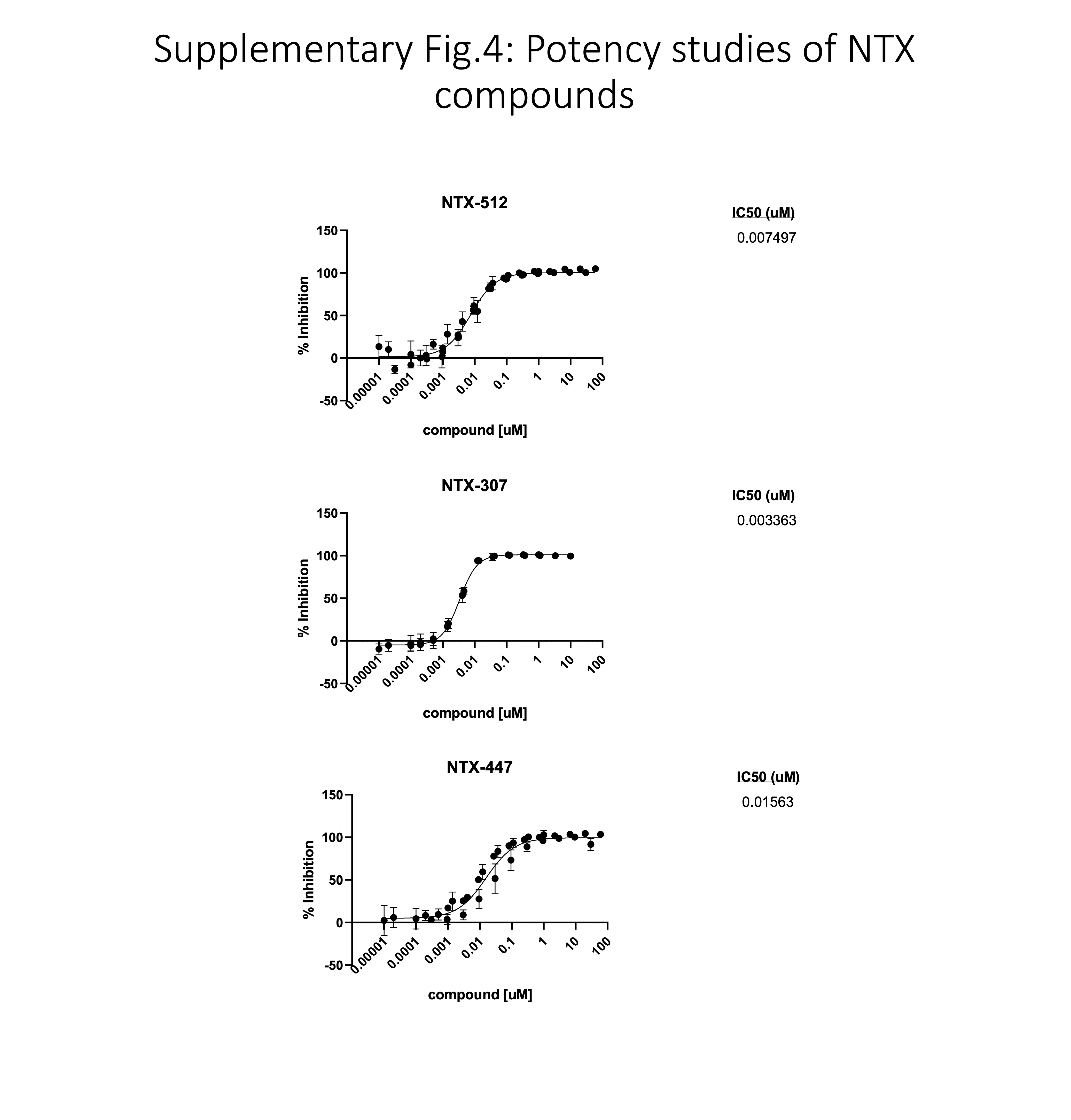

Supplement: Supplementary Figure 4 — Potency studies of NTX compounds. The potency of NTX compounds to inhibit Cbl-b was assessed by time-resolved measurement of fluorescence with fluorescence resonance energy transfer technology (TR-FRET). The figure shows dose-response curves and IC50s calculated using a 4-parameter dose-response equation. The X axis of the graphs show concentrations (µM) in log10. The Y axis show the responses (Cbl-b inhibition) expressed as percentages. Data was normalized using high and low assay controls: % Inhibition =100-(100*[(high control) - unknown)/(high control - low control)]. [file Image_4.jpeg]

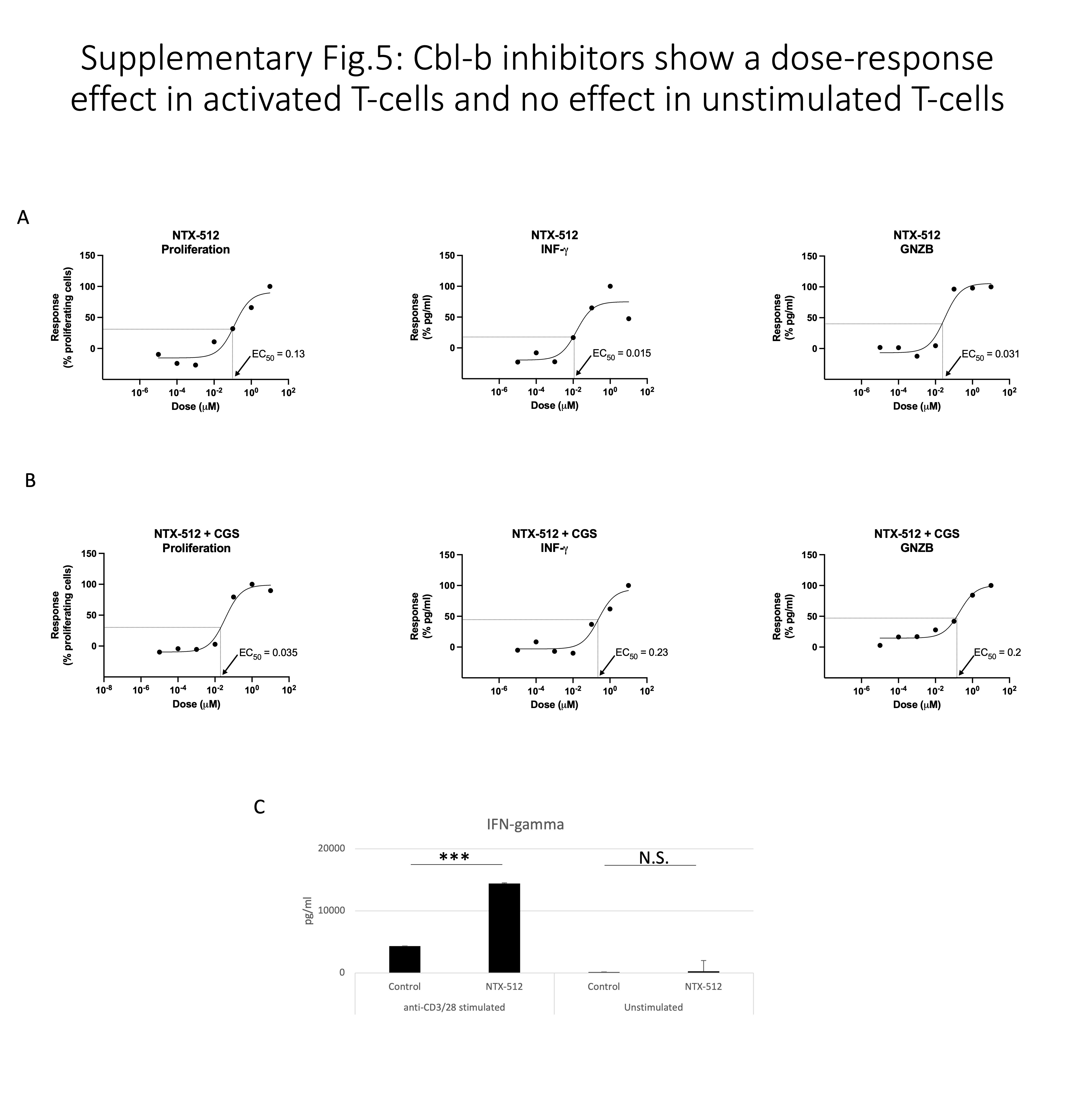

Supplement: Supplementary Figure 5 — Cbl-b inhibitors show a dose-response effect in activated T-cells and no effect in unstimulated T-cells. Dose-response curves and EC50s of proliferation and production of IFN-γ and Granzyme B (GNZB) in primary activated CD8+ T-cells isolated treated with different concentrations of (A) NTX-512 alone or against (B) 1µM CGS-21680 (CGS). The X axis of the graphs show concentrations (µM) in log10. The Y axis show the responses (proliferation or cytokine production) expressed as percentages. For clarity, the highest response value was set to 100% and the other values were set accordingly. (C) Production of INF-γ in activated or unstimulated primary CD8+ T-cells and treated with 1µM NTX-512 or vehicle-treated (Control, DMSO). The graphs show averages ± standard deviation from three independent experiments. ***p<0.001. two tailed T-test with equal variance. N.S, non-significant. [file Image_5.jpeg]

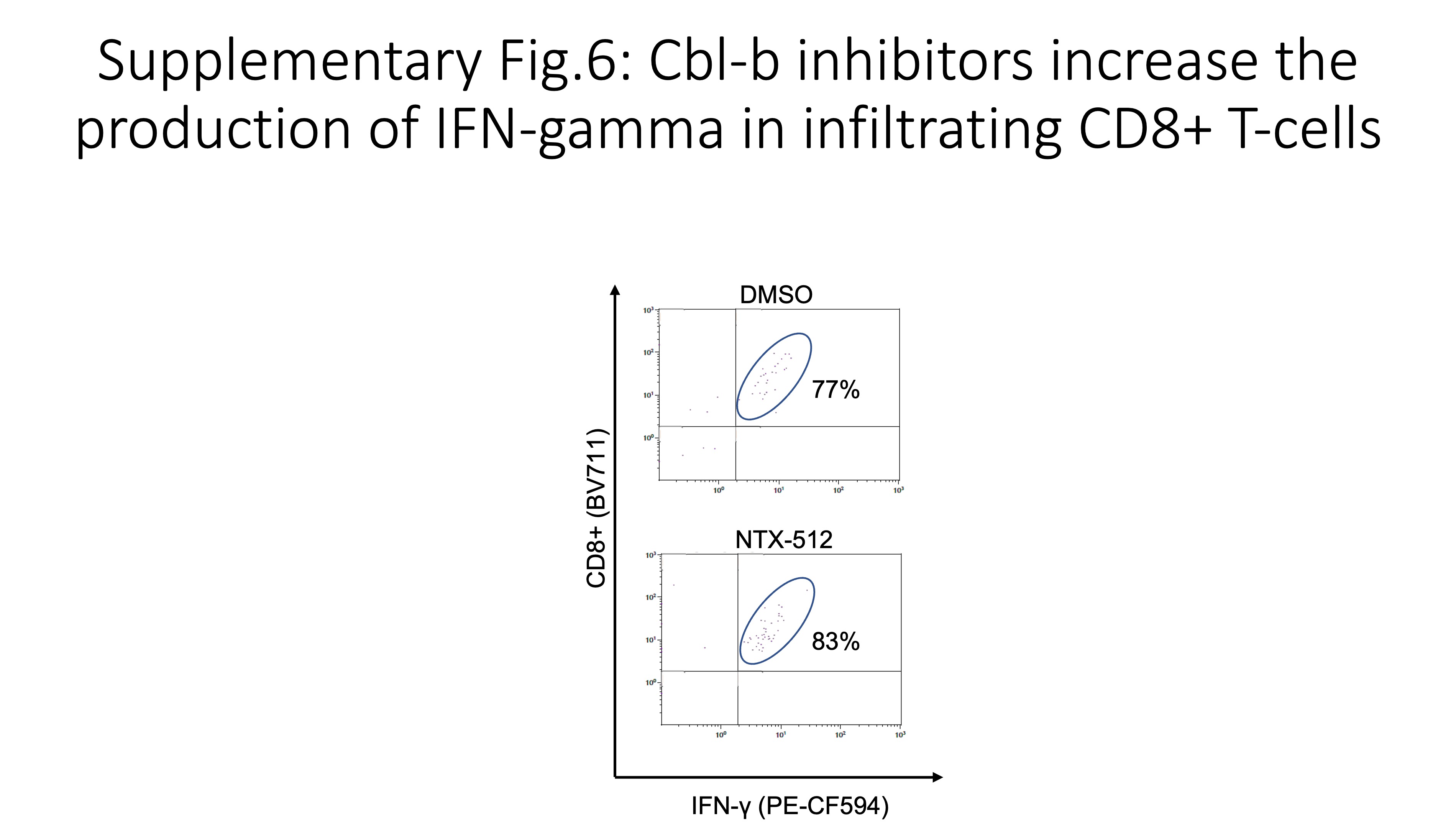

Supplement: Supplementary Figure 6 — Cbl-b inhibitors increase the production of IFN-gamma in infiltrating CD8+ T-cells. Percentage of IFN-gamma+ CD8+ T-cells in organoids untreated (DMSO) or treated with NTX-512. IFN-gamma+ CD8+ T-cells were gated for live cells, CD45+ and CD3+. [file Image_6.jpeg]

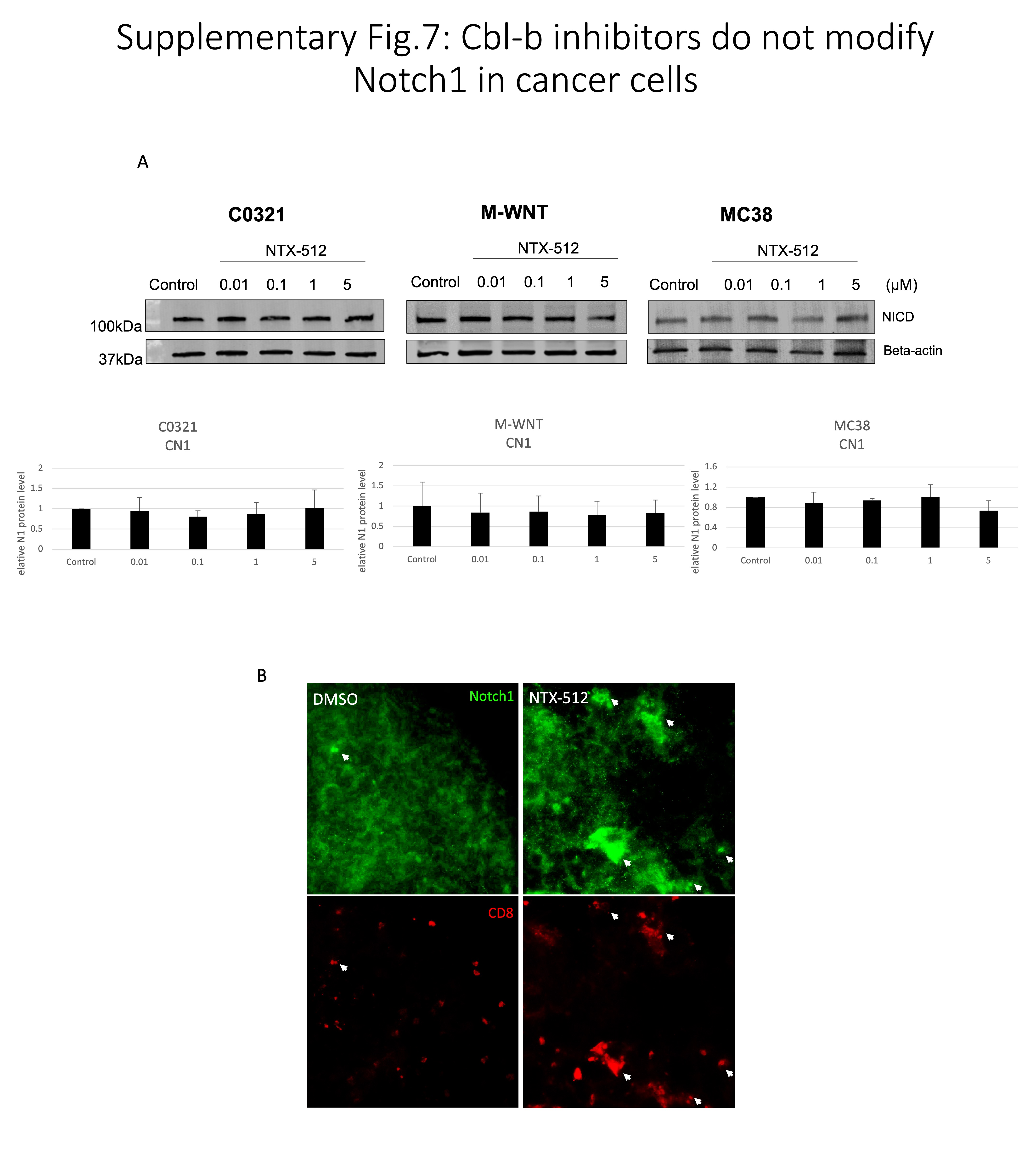

Supplement: Supplementary Figure 7 — Cbl-b inhibitors do not modify Notch1 in cancer cells. (A) Protein levels of Notch1 in TNBC cancer cell lines, C0321, M-WNT, and colon cancer, MC38, treated with vehicle (control, DMSO) or 0.01, 0.1, 1 and 5µM of NTX-512. The graphs show the densitometry analysis of NICD normalized by β-actin. The graphs show averages ± standard deviation from three independent experiments. Two tailed T-test with equal variance identify no significant differences in NICD in the samples. (B) Notch1 and CD8+ T-cell staining in C0321 organoids treated with vehicle (control, DMSO) or NTX-512. White arrows indicate co-localization of Notch1 and CD8+ T-cells. This panel show the images presented in without threshold adjustment to show Notch1 staining in surrounding cancer and stroma cells in organoids. [file Image_7.jpeg]
